# Measuring recovery after open lower limb fractures: combined objective functional tests and global perceived recovery outperform narrower metrics and a standard generic patient reported outcome measure

**DOI:** 10.1186/s12891-021-04356-9

**Published:** 2021-06-12

**Authors:** Ian Pallister, Gregory Jack Handley, Sharon Maggs, Ann-Marie Davies, Amanda Kyle, Owen Bodger, Hywel Dafydd

**Affiliations:** 1 Department of Trauma & Orthopaedics, Swansea University Medical School, c/o Morriston Hospital, Swansea, SA6 6NL UK; 2 Department of Surgery, Morriston Hospital, Swansea, UK; 3 Department of Physiotherapy, Morriston Hospital, Swansea, UK; 4 Department of Occupational Therapy, Morriston Hospital, Swansea, UK; 5 Senior Lecturer Statistician, Swansea University Medical School, Swansea, UK; 6 Department of Wales Centre for Burns and Plastic Surgery, Morriston Hospital, Swansea, UK

**Keywords:** Open lower limb fractures, Outcome, Recovery, Rehabilitation, Function, Complications

## Abstract

**Background:**

Open lower limb fractures are serious injuries requiring combined ortho-plastic surgery and have significantly worse outcomes than similar closed fractures. There is little objective published data to determine which functional outcome measures best reflect progress or completeness of physical recovery.

Our hypothesis was that objective measures combining strength, mobility and balance would better reflect recovery than isolated parameters (e.g. range of motion ROM) and would compare well to patients’ perceived recovery.

**Methods:**

Adult open lower limb fracture patients were reviewed 6 and 12 weeks, 6, 9 and 12 months post-injury. The mechanism, injury pattern, age, gender and treatment were recorded. Isolated parameter objective functional outcome measures (OFOMs) (ROM and MRC strength grade) were compared to combined OFOMs (timed up and go, comfortable gait speed and fast gait speed, Edgren Side Step Test (TUAG, CGS, FGS, ESST) and Single Leg balance. Patient reported outcomes were recorded (Global Perceived Effect (GPE) score and Disability Rating Index (DRI)). Statistical analysis used non-parametric tests (e.g. Spearman correlation) compared each with time since injury.

**Results:**

Sixty-eight patients (54 male) with a median age of 45(20–75) years. Of the 19 isolated OFOMs, only knee flexion and ankle plantar flexion ROM and strength improved with time (Spearman correlation *p* = 0.042, 0.008, 0.032, 0.036 respectively). TUAG, ESST, CGS, FGS and GPE scores showed significant improvement (Spearman correlation *p* < 0.001). Patients’ estimation of recovery paralleled these measures (Spearman correlation *p* < 0.001) with all but 2 patients achieving the minimum clinical important difference in DRI by 12 months compared to baseline. However, the GPE score had a higher proportion of improving responses than DRI at each time-point.

**Discussion:**

Functional recovery is a key determinant in patients returning to work, providing for themselves and their family or resuming independent living for older patients. This study has demonstrated time-related improvements in combined OFOMs measuring mobility, strength, agility and balance paralleling patients’ perception of recovery in the 12 months after open lower limb fractures. Over the same time-frame, the simple GPE score compared favourably with the DRI. Such parameters could become part of a defined core outcomes set. Focussing rehabilitation towards these combined OFOMs may help hasten recovery.

**Trial registration:**

South West Wales REC 06/WMW02/10).

## Background

Open lower limb fractures are serious and potentially life-changing injuries, often requiring combined orthopaedic and plastic surgical management in specialist units [1, 2]. Following surgery, rehabilitation to minimise swelling, scarring and promote independent mobility is vital. Even when the limb is preserved and wound and bone healing are complete, a wide range of clinical outcomes are seen, ranging from pain free mobility and a return to manual work, to ongoing pain, troublesome disfiguring scarring and problems with mobility. This distinguishes recovery from open lower limb fractures from their closed counterparts. Indeed, Disability Rating Index (DRI) scores at 12 months post injury are distinctly worse in open fracture patients compared to those with closed fractures [3, 4]. In our recent systematic review, we have shown that published outcome measures after either open and closed tibial fractures are inconsistently reported and seldom include direct measures of the patients’ locomotor ability [5]. For these reasons the need for a defined core outcome set for open lower limb fractures has been recognised [5]. This would enable the meaningful comparison of surgical strategies within and between future studies.

In recent years, interest has grown in the value of Patient Reported Outcomes Measures (PROMS) as distinct from the measures of technical success such as wound or bone healing, alignment and the number and complexity of reconstructive surgical procedures. However, there is little objective published data to determine which functional outcome measures best reflect either patient progress or completeness of their physical recovery.

As part of a larger project studying recovery from open lower limb fractures, the potential benefits of functional recovery measures that reflect strength, agility and endurance became apparent (Wales Lower Limb Trauma Recovery Project (WaLLTR) South West Wales Research Ethics Committee 06/WMW02/10) [6, 7]. These measures have been studied extensively in stroke rehabilitation, recovery from hip fracture and also sports medicine [8–11]. Given the paucity of data with respect to these combined measures in the open lower limb fracture population, a pragmatic decision was required to select a range of measures to be studied [12].

An observational study was conducted of the objective functional outcome measures recorded in adult open lower limb fracture patients attending the Morriston Hospital Ortho-Plastic Trauma Research Clinic (OPRC) and these findings were correlated with the passage of time from injury and the Disability Rating Index (DRI) [5, 6, 13, 14].

The aim of the study was to determine whether objective functional outcome measures (OFOMs) mirrored changes in health-related quality of life patient reported outcome measures with the passage of time. Our hypothesis was that measures of combined strength, agility and balance would better reflect recovery than isolated parameters such as range of motion.

## Methods

### Data source

Adult patients (aged 16–80) were identified during their recovery from open lower limb fractures which had required Ortho-Plastic management in Morriston Hospital, Swansea UK. They were approached and enrolled in the observational WaLLTR Study after informed consent was obtained. Follow-up in OPRC was scheduled at 6 and 12 weeks, 6, 9 and 12 months post-injury.

In all, 79 patients enrolled although 2 patients subsequently withdrew their consent and a further 9 patients did not attend the OPRC and were lost to research follow-up. The remaining 68 patients had sustained 70 open lower limb fractures and were enrolled from 15/04/2014 to 25/08/2017 and followed up for 12 months.

### Isolated outcome measures

In total 19 isolated OFOMs were recorded, comprising active and passive range of movement, and MRC strength grade at the hip, knee and ankle [15–17].. Range of motion (ROM) was measured using the following methods.

To measure passive ROM in the hip, with the patient lying supine, the hip was passively flexed and a goniometer positioned, centred on the greater trochanter, with the static arm horizontal and the dynamic arm positioned centrally along the lateral border of the femur. Active ROM was measured while standing, with the goniometer positioned and aligned in the same manner and the patient actively flexed their hip joint.

Passive ROM in the knee was measured with the patient lying prone. The examiner passively flexed the patients’ knee and used a goniometer centred on the lateral joint line of the knee, with the static arm horizontal and the dynamic arm positioned centrally along the lateral border of the tibia. Active knee ROM was measured with the patient sitting, the goniometer was centred on the lateral joint line of the knee, with the static arm horizontal, positioned centrally along the lateral border of the femur and the dynamic arm positioned centrally along the lateral border of the tibia, whilst the patient actively flexed their knee joint.

Passive ankle ROM was measured with the patient lying supine and the knee extended, the ankle was passively flexed by the examiner with the goniometer centred on the lateral malleolus with the static arm horizontal, positioned centrally along the lateral border of the tibia and the dynamic arm positioned centrally along the lateral border of the foot. This was repeated with the knee in 30° of flexion. Active ankle ROM was measured with the patient standing, utilising the knee-to-wall principle, in which the subject performed a weight-bearing lunge. The patient placed the test foot on a tape measure perpendicular to the wall and lunged forward so the knee touched the wall. The foot was moved away from the wall until the knee could only make slight contact with the wall while the foot remained flat on the ground. This position placed the ankle in maximal dorsiflexion, and the distance from the great toe to the wall was measured in centimetres, with each centimetre corresponding to approximately 3.6° of ankle dorsiflexion. If the patient was unable to touch knee-to-wall with the great toe in contact with the wall, the distance between the knee and the wall is measured, in centimetres and was recorded as a negative value.

### Combined outcome measures

In addition to the isolated measures a battery of combined functional measures were recorded, including combined strength, agility and balance.

The “Timed Up & Go” (TUG) Test was recorded as the time taken to stand up from a seated position in a chair, walk straight for 3 m, turn 180°, walk back to the chair, then sitting down [10]. Completing this exercise in 10 s or less is regarded as normal mobility, 30 s or more reflecting significantly impaired mobility. The Comfortable Gait Speed (CGS) and Fast Gait Speed (FGS) were determined by measuring the walking speed in the middle 10 m of a 14 m straight line after asking the participant to “Walk at your preferred walking speed as if you were walking in a park” (CGS) and “Walk as fast and as safely as you can” (FGS) respectively [9].

The Edgren Side Step Test (ESST: metres) quantifies an individual’s mobility or agility in the lateral direction. Four cones are placed in a line each 1 m from its neighbour [11]. Starting at the centre cones, the patient was instructed to side step to the left until their left foot had touched or crossed the left outside cone or tape mark but not to cross their feet. Then, they were asked to sidestep to the right until their right foot had touched or crossed the outside cone or tape mark. The patient sidestepped back and forth to the outside cones as rapidly as possible for 10 s. The patient was given one point per completion of each 1 m increment marked by a cone or tape mark. If the far end lines were not reached, those points were not awarded. A score of 0 was given if they failed to keep their trunk and feet pointing forward at all times or crossed their legs.

Balance as a discrete parameter was assessed using the Single Leg Stand Test (SLS: seconds) [18]. The length of time for which a participant balanced on the required leg (without the other lower limb touching and with their arms by their sides) was recorded, up to a maximum of 30 s.

### PROMS

The third set of ourcomes we collected were patients reported outcome measures.

The Global Perceived Effect score was recorded on a 0-100 mm linear scale, with none of their previous scores being visible to the patient. It was explained that the scale represented them at their very worst immediately following their injury (zero), to being restored to completely back to their former selves (100 mm) [19–22].

The Disability Rating Index (DRI) is a validated questionnaire which was completed by the patient at each timepoint. It consists of 12 items which specifically relate to lower limb function. These are combined to give a score from zero (no disability) to 100 (complete disability). The minimum clinically important difference (MCID) in the DRI is 8 points [3, 14] .

### Data analysis

The data set comprised all measurements from all three categories (isolated, combined and PROM). Data completeness was fairly good although not all values were available for all follow up visits and the number of follow up visits varied between patients. The sample size for each hypothesis test is given in the tables.

Additional variables were extracted from the raw data, such as the classification of each follow up interval to indicate whether the patients appeared to have improved, stayed the same or worsened, according the a given test.

We also used the time of each follow up and the time since injury to represent progress along the journey to recovery.

Statistical analysis was mostly performed in SPSS version 25 and primarily consisted of non-parametric tests (Spearman correlation) to explore the correlations between the measures and the time elapsed since injury. Non-statistical, visual methods were also utilised to illustrate the data series.

It was also necessary to employ the use of of methods to test for differences between measurements of correlation so as to determine whether some tests performed significantly better than others. Such a comparison of correlations is not often performed and it was necessary to employ specialised methods using the ‘R’ package. Additional algorithms obtained from https://www.ibm.com/support/pages/differences-between-correlations were coded into R (OB) [23, 24].

## Results

### Data summary

The data set consisted of 68 patients. Fifty-four of these were male with a median age of 45 years (range 20–74). Fifteen patients were female with a median age of 51 years (range 22–73). The mechanism of injury, AO fracture and Gustilo-Anderson classification are summarised in Table 1. The type of definitive fracture fixation and soft tissue repair is shown in Table 2.

**Table 1:** Mechanism of Injury, AO/OTA Fracture Classification and Gustilo-Anderson Open Fracture Grade

Mechanism of Injury		AO/OTA Fracture Type		Gustilo-Anderson Classification	
Crush Injury	5	Femoral Shaft 32	2	I	13
Occupant RTC	3	Tibia- Proximal metaphysis 41	5	II	-
Motorcycle RTC (Bicycle)	14 (1)	Tibial Shaft 42	34	IIIA	11
Pedestrian RTC	11	Tibia- Distal metaphysis 43	14	IIIB	44
Fall from Height	14	Ankle 44	13	IIIC	1
Fall from Standing	11

**Table 2:** Method of operative fracture fixation and mode of soft tissue closure or reconstruction

Method of Fixation		Mode of Soft Tissue Cover	
CIRCULAR FRAME	8	Primary Closure	13
Intramedullary Nail(Hind Foot Nail)	37 (1)	Split Skin Grafting only	18
ORIF	23	Fasciocutaneous Flap	18
Amputation	1	Local Muscle Flap	9
Masquelet	1	Free Tissue Transfer	16
Transport	1	Papineau	1

The most common mechanism of injury was due to road traffic collisions, followed by falls from a standing height. Crush injuries were sustained by 5 individuals. The Gustilo-Anderson Grade was determined definitively at the time of surgical treatment with Type I 13, IIIA 11, IIIB 44, IIIC 1. The soft tissue injury associated with the IIIC injury warranted only split skin grafting. The commonest location of the open fracture was tibia shaft (34/68) and the most frequently employed means of definitive stabilisation was an intramedullary nail (37/68). Thirteen patients had wounds suitable for primary closure, 27 were treatable using local flaps and 16 required reconstruction using free tissue transfer; one primary amputation was required. One patient was managed using open cancellous bone grafting (Papineau technique) and another using induced membrane formation (Masquelet technique) [25, 26].

One patient who had originally undergone intramedullary nail fixation and anterolateral thigh flap coverage required revision to a below knee amputation during the original admission due to flap failure and the lack of any suitable salvage procedure.

Three patients required revision intramedullary nailing at approximately 3, 7 and 11 months post injury for correction of alignment. Two had preceding problems with wound healing but none had evidence of deep infection on intraoperative cultures.

### Graphical methods

Most of the measures (in all three categories) showed signs of improvement across the follow up period. However for a measurement to be a useful guide to recovery the size of the ‘signal’ (the recovery of the patient) should be large compared to the ‘noise’, or the volatility of the measurements being used. This varied dramatically between different methods with many showing only gradual improvement across the recovery period.

**Fig. 1:**
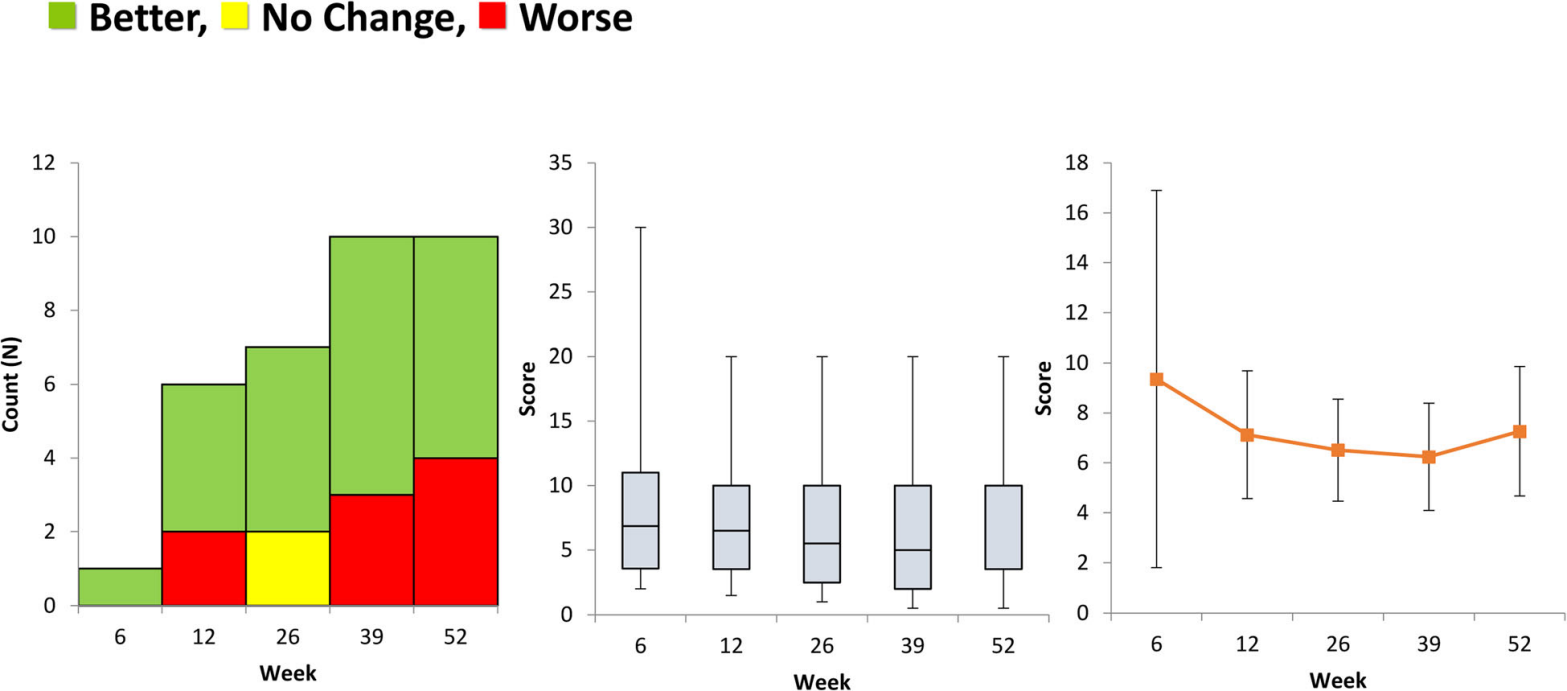
Active Ankle Dorsiflexion- An Isolated Objective Functional Outcome Measure

**Fig. 2:**
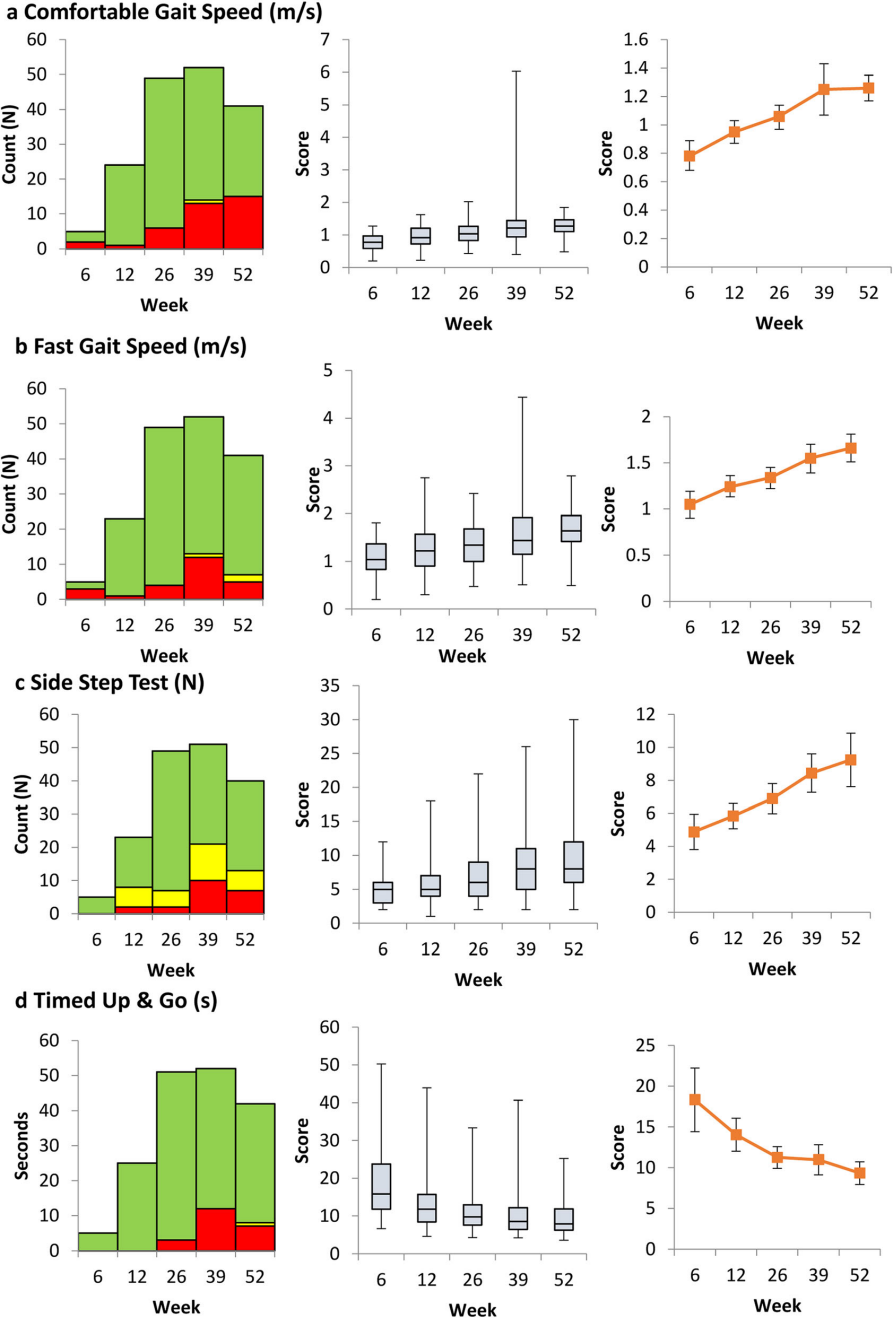
Combined Objective Functional Outcome Measures  Better,  No Change,  Worse 2a Comfortable Gait Speed (m/s)

Five of best performing methods have been visually represented in Figs. 1 and 2. Each method is displayed in three different ways. The first graph shows whether the patient appears to have improved since the previous visit. To be an effective way of assessing progress a measurement improve steadily and predictably. The second graph is a Boxplot showing inter-patient variation in the measure. A method with low inter-patient variation would be more objective in assessing recovery. The third graph shows the mean value, plus confidence interval, for the measure at each time point. Ideally the improvement should large compared to the size of the confidence interval.

### Isolated outcome measures

Each of the 19 different isolated measures was tested to determine if it correlated with the time that had elapsed since treatment. Only 4 showed a significant level of correlation. In order of correlation strength these were Ankle Plantar Flexion Passive ROM (rho = 0.166, *p* = 0.008), Knee Flexion Strength (rho = 0.138, *p* = 0.032), Ankle Plantar Flexion Strength (rho = 0.134, *p* = 0.036) and Knee Flexion Active ROM (rho = 0.129, *p* = 0.042). A comprehensive list of all of these results can be found in Table 3.

**Table 3:** Isolated objective outcome measures: active and passive range of motion, mrc grade strength – spearman correlation versus time

	Measure	Spearman correlation (raw)	Spearman Correlation (abs)	N	CI (95%)	***p***-value
Range of Motion	Hip Flexion Active	0.010	0.010	245		0.879
	Hip Flexion Passive	-0.050	0.050	254		0.424
	Hip Extension Active	0.061	0.061	157		0.452
	Hip Extension Passive	0.068	0.068	184		0.234
	**Knee Flexion Active**	0.129*	**0.129**	**252**	**0.006,0.248**	0.042*
	Knee Flexion Passive	-0.023	0.023	252		0.712
	Knee Extension Active	-0.414	0.414	7		0.356
	Knee Extension Passive	0.175	0.175	26		0.392
	Ankle Plantar Flexion Active	0.142	0.142	252		0.025*
	**Ankle Plantar Flexion Passive**	0.166**	**0.166**	**252**	**0.044,0.283**	0.008**
	Ankle Dorsiflexion Active	-0.078	0.078	87		0.475
	Ankle Dorsiflexion Passive	-0.005	0.005	119		0.960
Strength	Hip Flexion Strength	-0.014	0.014	250		0.825
	Hip Extension Strength	0.067	0.067	240		0.299
	Hip Abduction Strength	0.060	0.060	243		0.353
	**Knee Flexion Strength**	0.138*	**0.138**	**244**	**0.013, 0.259**	0.032*
	Knee Extension Strength	0.064	0.064	252		0.312
	**Ankle Plantar Flexion Strength**	0.134*	**0.134**	**246**	**0.010, 0.254**	0.036*
	Ankle Dorsiflexion Strength	0.112	0.112	238		0.086

Although statistically significant these correlations are considered to be “very weak”. Given the relative weakness of these isolated measures only the best performing of these (Ankle Plantar Flexion Passive ROM) will be retained for comparison with other methods. These weak results are apparent in Fig. 1, which shows only gradual improvement and frequent regression.

### Combined outcome measures

The combined tests performed much better than the isolated tests, with all 4 of them reaching a high level of statistical signifiance in their correlation with time (*p* < 0.001 in all cases). This is indicative of much higher levels of correlation, with TUAG (rho = 0.388), ESST (rho = 0.354) and FGS (rho = 0.352) being classified as “weak” and CGS (rho = 0.431) as “moderate” correlation (Table 4).

**Table 4:** Combined objective functional outcome measures and patient reported outcome measures - spearman correlation versus time

Measure	Spearman correlation (raw)	Spearman Correlation (abs)	N	CI (95%)	***p***-value
Timed Up & Go (TUAG)	-0.388	0.388	272	0.283, 0.484	<0.001***
Edgren Side Step Test (ESST)	0.354	0.354	261	0.244, 0.455	<0.001***
Comfortable Gait Speed (CGS)	0.431	0.431	264	0.328, 0.524	<0.001***
Fast Gait Speed (FGS)	0.352	0.352	264	0.242, 0.453	<0.001***
Global Perceived Effect (GPE)	0.518	0.518	296	0.430, 0.596	<0.001***
Disability Rating Index (DRI)	-0.430	0.430	233	0.320, 0.529	<0.001***

Figure 2 shows how much more effective these measures are likely to be, with stable and consistent improvement shown through time relative to the variation between individuals.

### PROMS

The final group of measurements were the patient reported outcome measures. These also showed good promise, with Global Percieved Effort (rho = 0.518, *p* < 0.001) and Disability Rating Index (rho = 0.430, *p* < 0.001) both being significantly correalted with recovery. GPE showed “moderate” correlation and the highest correlation coefficient observed in this study.

**Fig. 3:**
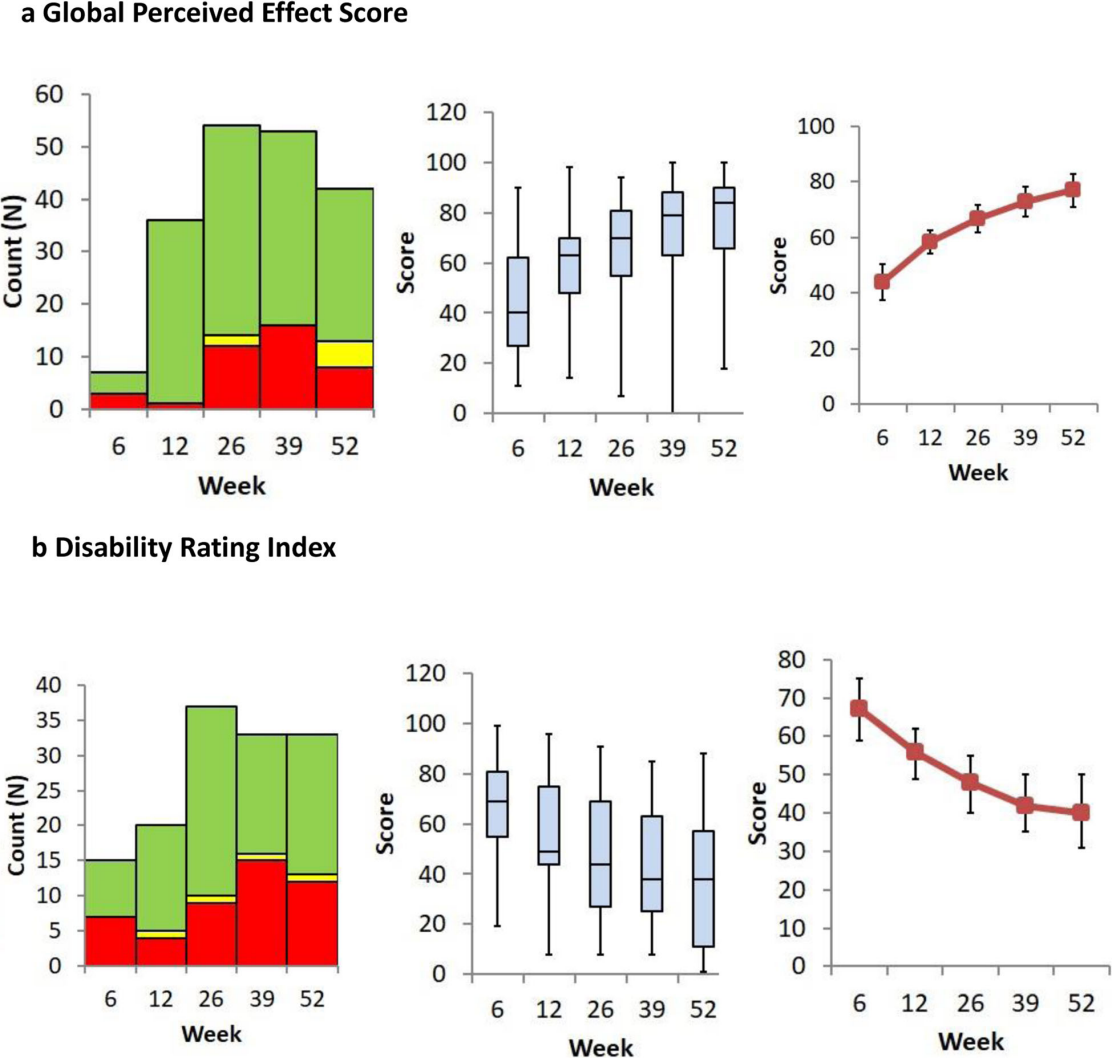
Changes in Patient Reported Outcome Measures  Better,  No Change,  Worse, 3a Global Perceived Effect Score

The DRI minimum detectable change has been recognised as +/− 2.7 and so “no change” in this figure reflects responses within this range rather than being simply numerically equal. All but 2 patients DRI score had improved by the MCID of 8+ points between the baseline and 12 month assessments. However, the GPE score compared favourably with the DRI, with a higher proportion of improving responses at each time-point (Fig. 3).

### Comparing methods

**Fig. 4:**
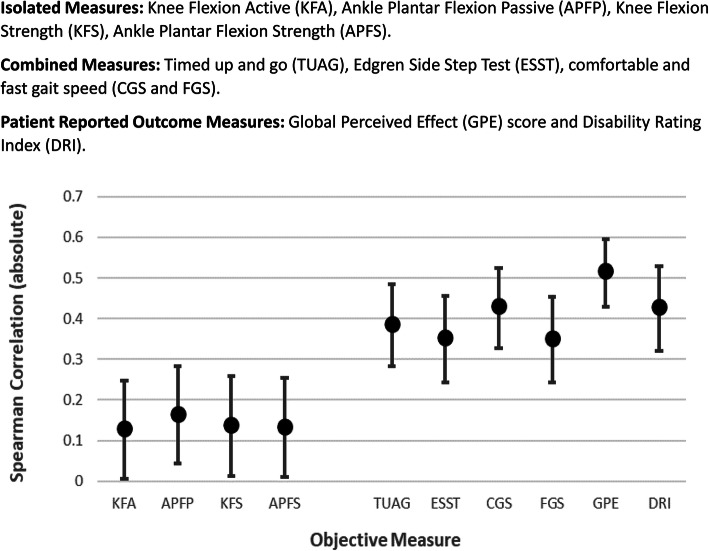
Correlation Between Isolated and Combined Objective Functional Outcome Measures. Isolated Measures: Knee Flexion Active (KFA), Ankle Plantar Flexion Passive (APFP), Knee Flexion Strength (KFS), Ankle Plantar Flexion Strength (APFS). Combined Measures: Timed up and go (TUAG), Edgren Side Step Test (ESST), comfortable and fast gait speed (CGS and FGS). Patient Reported Outcome Measures: Global Perceived Effect (GPE) score and Disability Rating Index (DRI)

In total only 10 measures were significantly correlated with time, and therefore recovery. These are summarised in Table 5. A visual comparison of the (absolute) correlation coefficients, including the confidence interval of measurement is given in Fig. 4. While the combined measures and PROMs appear distinctly better than the isolated measures, statistical testing was performed to formally rank the measurement.

**Table 5:** Comparison of the correlations between different Measures

Test 1	Comparison	Test 2	N or N1/N2	***p***-value	Reference
TUAG	>	APFP	272/252	0.006**	As described by Glass (1996) [23]
ESST	>	APFP	261/252	0.022*	
CGS	>	APFP	264/252	0.001**	
FGS	>	APFP	264/252	0.024*	
GPE	>	APFP	296/252	0.000***	
DRI	>	APFP	233/252	0.001***	
DRI	.	TUAG	233/272	0.574	
DRI	.	ESST	233/261	0.322	
DRI	.	CGS	233/264	0.989	
DRI	.	FGS	233/264	0.308	
DRI	.	GPE	233/296	0.197	
TUAG	.	ESST	261	0.306	As described Sheshkin (2004) [24]
TUAG	.	CGS	263	0.302	
TUAG	.	FGS	263	0.057	
TUAG	.	GPE	265	0.193	
ESST	.	CGS	257	0.071	
ESST	.	FGS	257	0.469	
ESST	<	GPE	255	0.033*	
CGS	>	FGS	263	0.004**	
CGS	.	GPE	257	0.198	
FGS	<	GPE	257	0.011*	

**Isolated Measure**: Ankle Plantar Flexion Passive (APFP)

**Combined Measures**: Timed up and go (TUAG), Edgren Side Step Test (ESST), comfortable and fast gait speed (CGS and FGS). PROMS: Global Perceived Effect (GPE) score and Disability Rating Index (DRI)

Using the methods described by Glass (1996) and Sheshkin (2004) [23, 24] > indicates Test 1 has a greater correlation with time than Test 2,whereas < indicates Test 2 correlates better with time. N or N1/N2 are the sample sizes (i.e. how many correlations were used). N used paired correlations and N1/N2 used two independent sets of correlations, hence two sample sizes

Pairwise comparisons were made between the correlation coefficients recorded for each measure to determine whether the differences were statistically significant. These results are given in Table 5. Unsurprisingly all combined measures and PROMs performed significantly better than the Ankle Plantar Flexion Passive ROM. There were relatively few significant differences between the remaining tests, with Comfortable Gait Speed and Global Perceived Effect appearing to be slightly better than some of the other measures.

## Discussion

In this observational study of 68 adult, post open lower limb fracture patients, of the 19 isolated OFOMs, only knee flexion and ankle plantar flexion ROM and strength improved with time (Spearman correlation *p* = 0.042, 0.008, 0.032, 0.036 respectively). TUAG, ESST, CGS, FGS and GPE scores showed significant improvement (Spearman correlation *p* < 0.001). Patients’ estimation of recovery paralleled these measures (Spearman correlation *p* < 0.001) with all but 2 patients achieving the minimum clinical important difference in DRI by 12 months compared to baseline. However, the GPE score had a higher proportion of improving responses than DRI at each time-point. This study has demonstrated time-related improvements in combined OFOMs measuring mobility, strength, agility and balance paralleling patients’ perception of recovery in the 12 months after open lower limb fractures. Over the same time-frame, the simple GPE score compared favourably with the DRI.

We hypothesised that improvements in objective outcome measures combining strength, agility and balance would mirror patients’ own perceptions of their recovery after open lower limb fractures. There is a paucity of published data to establish reliable core clinical outcome measures in this group of patients [5, 27]. Open lower limb fractures can be life-changing injuries. Even when complication rates are low, a recent randomised multi-centre study has shown significant levels of patient reported disability throughout the first 12 months post-injury [3, 28, 29]. In this study of 427 patients who completed the trial, DRI scores improved from the mid-sixties at 3 months to mid-forties at 12 months. By comparison, the DRI score of patients with closed distal tibial fractures were distinctly better with average scores in the low 20s, around double the MCID for this measure [4].

Qualitative research has thrown light on patients’ experiences and perceptions of the impact of open lower limb fractures, which extend far beyond the obvious physical consequences [3, 6, 28]. Nevertheless, functional recovery is the key determinant in patients being able to return to work and provide for themselves and their family or resume independent living for older patients. A painless limb free of contractures may lack the strength, proprioception and endurance necessary to undertake everyday activities. This study has demonstrated a clear relationship between combined measures of strength, agility and balance with patient reported measures of recovery and health-related quality of life collected in parallel in the 12 months after open lower limb fractures. Focussing rehabilitation towards improvement in these combined measures may help hasten recovery.

Isolated parameter objective functional outcomes measures did not generally show consistent improvement with the passage of time. In a previous observational study, ankle dorsiflexion at the time of cast removal in post-ankle fracture patients has been associated with better recovery quantified by Olerud and Molander ankle score, the Lower Extremity Functional Scale and also the Global Perceived Effect score (GPE) 6 weeks and 6 months later [16]. While the lunge weight-bearing method to measure dorsiflexion may be very reliable [15], it cannot be used effectively in those unable to fully weight-bear. In this study, patients with articular fractures (AO 43B/C and AO 44 *n* = 22) were advised to be non-weight-bearing for 6 weeks and then incrementally increase to be fully weight-bearing by 12 weeks.

Gait speed is a recognised form of assessment in patients following stroke and hip fracture, with faster gait speeds associated with greater degrees of independence and mobility [30]. Speeds greater than 1.2 m/s are considered normal, 0.8–1.2 m/s community ambulators, 0.4–0.8 m/s limited community ambulators and walking speeds lower than this leading to the patient essentially being housebound. In Fig. 2a it can be seen that by 9 months, approximately 75% of the patients completing the assessment were comfortable walking at a pace of 1 m/s or faster.

Comfortable and maximum gait speeds have been assessed in healthy individuals and stratified according to gender, height and age [9]. Gender had little demonstrable effect, however gait speed did correlate with muscle strength, in particular hip abductors for comfortable gait speed and knee extensors for maximum gait speed. Comfortable gait speed declined slowly with increasing age from 20th to 70th decades (mean 1.4 m/s – 1.3 m/s) and maximum gait speed more sharply (mean 2.5 m/s – 1.9 m/s). In this study population, Fig. 2b shows that by 6 months approximately 75% of patients completing the assessment achieved a fast gait speed of 1 m/s or greater. By 12 months post injury, over half the patients could sustain a speed of 1.5 m/s. At 12 months, only 9 patients had TUAG times greater than 10 s, indicating normal mobility as determined by this test, although none exceeded 18 s.

Of the combined objective outcome measures, improvements in comfortable gait speed appear to have the strongest relationship with the passage of time after injury. Perhaps the request to walk at their own comfortable pace elicits a more natural and instinctive response than the other measures in which the patient may wish to push themselves to perform, but also feel apprehensive about doing so.

In terms of patient reported outcome, the use of the GPE scale in the manner described yielded responses specific to the injury itself, inviting comparison of themselves at their worst immediately after injury versus how they were at their best beforehand. The responses elicited using the DRI gave medians comparable to those reported previously (approximately 60 at 3 months recovering to 40 at 12 months) but with a wide range of responses. The lack of consistent improvement with time for individual patients suggests that this score may be better restricted to studies of populations at defined and well separated timepoints, rather than being used as a “monitoring” score with serial measurements taken only a few months apart.

This study has limitations. Not all patients sustaining lower limb fractures will have been identified for potential enrolment. A very small proportion of those approached declined consent to attend the OPRC. Of those who did, few were able to attend every single appointment and so the data is inevitably incomplete. Nevertheless the results obtained are consistent across the population studied. No comparison has been made with recovery in patients with comparable closed fracture types. Available resources did not permit this, although the long-term goal of the project is to extend this approach to closed fracture patients. While such a comparison would have been of interest, published data and clinical experience would strongly suggest that these injuries are distinctly different in their outcomes, as well as surgical strategy [3, 4].

Determining outcomes in healthcare has moved beyond measuring the frequency of technical problems or achievements. Patients with deep infection or non-union after open fractures will require further management and so those complications may be better regarded not as outcomes, but events along the road to recovery. PROMS, even if derived from the specific patient population under review, will introduce an element of subjectivity. However patients’ perception of their recovery is essential in completing the picture [6, 7].

That said, in the same way that blood pressure can be measured in research into the treatment of hypertension, recovery following a limb-threatening injury can be assessed by objectively assessing the physical performance of the limb for its role, i.e. locomotion. Functional recovery is vital as this will determine independence, resumption of caring for their family and return to work, all of which are so important in sustaining an individual’s self-worth. Identifying objective functional outcome measures which mirror patients’ perception of recovery has great value in helping to focus rehabilitation and forming part of a set of core clinical outcomes for future research.

The ideal outcome measures for these severe (and indeed many less severe) injuries are yet to be determined. In a recent workshop involving patients, their family members, researchers and clinicians, walking ability/mobility was identified as a key outcome after open lower limb fractures [31]. The objective measures described here were originally developed with patients whose mobility was impaired in mind. Further research is needed to refine these measures or develop new ones which reflect recovering mobility more precisely. In this way, a future core outcomes set will enable surgical strategies within and between studies to be meaningfully compared.

## Data Availability

The datasets used and/or analysed during the current study are available from the corresponding author on reasonable request.
